# Comparative Analysis of Chloroplast Pan-Genomes and Transcriptomics Reveals Cold Adaptation in *Medicago sativa*

**DOI:** 10.3390/ijms25031776

**Published:** 2024-02-01

**Authors:** Tianxiang Zhang, Xiuhua Chen, Wei Yan, Manman Li, Wangqi Huang, Qian Liu, Yanan Li, Changhong Guo, Yongjun Shu

**Affiliations:** 1 Key Laboratory of Molecular Cytogenetics and Genetic Breeding of Heilongjiang Province, College of Life Science and Technology, Harbin Normal University, Harbin 150025, China; hsdztx@stu.hrbnu.edu.cn (T.Z.); limanman1208@stu.hrbnu.edu.cn (M.L.); kaku3008@hrbnu.edu.cn (C.G.); 2 International Agriculture Research Institute, Yunnan Academy of Agricultural Sciences, Kunming 650200, China; cxh@yaas.org.cn; 3 Institute of Tropical and Subtropical Cash Crops, Yunnan Academy of Agricultural Sciences, Baoshan 678000, China; rjsyw@yaas.org.cn (W.Y.); rjslq@yaas.org.cn (Q.L.); 4 National Engineering Research Center for Ornamental Horticulture, Yunnan Flower Breeding Key Laboratory, Flower Research Institute, Yunnan Academy of Agricultural Sciences, Kunming 650200, China; hwq@yaas.org.cn

**Keywords:** alfalfa, chloroplast genome, population structure, genetic variation, cold stress

## Abstract

Alfalfa (*Medicago sativa*) is a perennial forage legume that is widely distributed all over the world; therefore, it has an extremely complex genetic background. Though population structure and phylogenetic studies have been conducted on a large group of alfalfa nuclear genomes, information about the chloroplast genomes is still lacking. Chloroplast genomes are generally considered to be conservative and play an important role in population diversity analysis and species adaptation in plants. Here, 231 complete alfalfa chloroplast genomes were successfully assembled from 359 alfalfa resequencing data, on the basis of which the alfalfa chloroplast pan-genome was constructed. We investigated the genetic variations of the alfalfa chloroplast genome through comparative genomic, genetic diversity, phylogenetic, population genetic structure, and haplotype analysis. Meanwhile, the expression of alfalfa chloroplast genes under cold stress was explored through transcriptome analysis. As a result, chloroplast genomes of 231 alfalfa lack an IR region, and the size of the chloroplast genome ranges from 125,192 bp to 126,105 bp. Using population structure, haplotypes, and construction of a phylogenetic tree, it was found that alfalfa populations could be divided into four groups, and multiple highly variable regions were found in the alfalfa chloroplast genome. Transcriptome analysis showed that tRNA genes were significantly up-regulated in the cold-sensitive varieties, while *rps7*, *rpl32,* and *ndhB* were down-regulated, and the editing efficiency of *ycf1*, *ycf2,* and *ndhF* was decreased in the cold-tolerant varieties, which may be due to the fact that chloroplasts store nutrients through photosynthesis to resist cold. The huge number of genetic variants in this study provide powerful resources for molecular markers.

## 1. Introduction

Chloroplasts are important organelles with autonomous genetic information within the cell [1]. Chloroplasts are involved in photosynthesis and energy conversion in plants and are commonly found in plants and algae [2]. The chloroplast genome is highly conserved in gene composition and structural arrangements [3,4]. Chloroplast genomes are structurally simple, have a relatively slow rate of evolution and low nucleotide substitution rates compared to nuclear genes, are mostly maternal in inheritance, and lack recombination [5,6]. Therefore, chloroplast genomes have been widely used for species identification and plant phylogenetic analysis [7,8]. Since the first tobacco chloroplast genome was determined in 1986, there have been thousands of species with chloroplast genome sequence data identified in the NCBI database [9]. Chloroplasts of higher plants have a typical quadripartite circular structure, and the size of the chloroplast genomes ranges between 120 kb and 180 kb and usually include 100–130 genes [10]. They consist of a pair of inverted repeats (IRs) with the same sequence in opposite directions, a large single copy region (LSC), and a small single copy region (SSC) [11,12]. During the long-term evolution, there also exists a class of chloroplast genomes missing the IR region in nature [13,14], such as the chloroplast genomes of some legumes, including *Trifolium subterraneum*, *Medicago truncatula*, and *Cicer arietinum*. This type of chloroplast genome lacks an IR region, called the inverted repeat lacking clade (IRLC) [15,16]. However, these chloroplast genomes are only about 120 kb, which also proves that the IR region is not an essential region of the chloroplast genome [17]. Chloroplast genome structure variation can be used to explore evolutionary relationships between species [18]. The sequences of coding and non-coding regions of the chloroplast genome can not only be used for phylogenetic analysis but also are ideal material for species identification [19].

Alfalfa (*Medicago sativa*) is known as the “*Queen of Forages*”, with a cultivating area of approximately 32 million hectares globally [20,21]. As one of the most important forage crops in the world, alfalfa has excellent characteristics, such as high protein levels, high digestibility, rapid growth, and strong environmental adaptability [22]. However, alfalfa has the characteristics of wide distribution and variable life-types, which makes it difficult for the academic community to directly correlate the evolutionary history of alfalfa with its geographic and habitat characteristics [22,23]. Chloroplast genes play an important role in plant adaptation to environmental changes and can quickly respond to environmental changes. Chloroplast genes actively involved in the cold stress response of plants by encoding proteins participate in photosynthesis, transcriptional regulation, and signal transduction [24]. Research has shown that low temperatures can inhibit the biogenesis and development of chloroplasts during the early greening stage of rice (*Oryza sativa* L.) seedlings [25]. *Atpl* and *atpE* play an important role in the adaptation of species to environmental changes within the *Celastraceae* family, affecting electron transfer and energy synthesis processes [26]. Tobacco lacking the chloroplast gene *petL* displays impaired photosynthesis and bleached leaves in cold conditions [27]. These studies show that the chloroplast genome can provide a great deal of genetic information that can be used as a basis for studying plant cold tolerance.

In recent years, chloroplast genomes have made important contributions in exploring the evolution of species and the relationships between species [14]. The chloroplast genomes of different subspecies of some plants have been sequenced. For instance, research on three subspecies of rice, including *Oryza nivara* [28], *Oryza sativa indica* [29], and *Oryza sativa japonica* [30], revealed their phylogeny and different domestication processes based on chloroplast genome analyses [31]. In modern phylogenetic studies, more and more species have been analyzed for chloroplast genomes with the increasing improvement of sequencing technologies, and studies on *Cucumis sativus* L. [32], *Fragaria ananassa* [33], and *Carya cathayensis* [34] have identified their origin, evolution, and genetic diversity. The alfalfa genome has now been extensively sequenced, and studies on 220 alfalfa germplasms [35] and 137 alfalfa global core germplasms [36] have been based on the genome, but few data from the chloroplast genome are available to explore inter-species and intra-species relationships. However, legumes have experienced a large number of chloroplast genome rearrangements, and the study of alfalfa chloroplast genomes is necessary as well as conducive to the study of alfalfa varieties for further differentiation.

In this study, 231 alfalfa chloroplast genomes were successfully assembled. The genetic diversity of the chloroplast genome of alfalfa was explored by constructing the chloroplast pan-genome and comparative genome, haplotype analysis, phylogenetic analysis, and genetic structure analysis. Meanwhile, transcriptome analysis was used to reveal the response of alfalfa chloroplast genes under cold stress. The results provide the foundation for the study of alfalfa chloroplast genomes, phylogenetic trees, genetic diversity, and adaptation of alfalfa to cold stress. 

## 2. Results

### 2.1. General Characteristics of 231 Alfalfa Chloroplast Genomes

**Figure 1:**
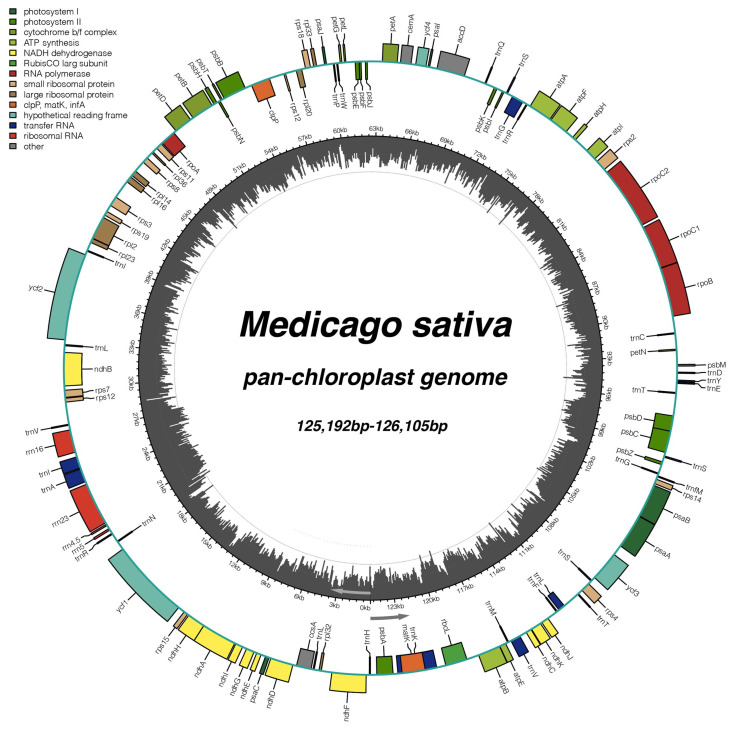
Pan-chloroplast genome map of alfalfa. Arrows indicate that genes outside the outermost circle are expressed in a counterclockwise direction, and genes inside the outermost circle are expressed in a clockwise direction. Different colored squares indicate genes with different functions; dark grey shading indicates GC content.

The chloroplast genome of *Medicago sativa* Deqin (MN218692) was selected as a reference genome, and the mapping reads were screened for assembly [37]. We successfully assembled and annotated a total of 231 chloroplast genomes by removing redundancy from 359 resequencing data (Figure S1). The sequence lengths of the 231 chloroplast genomes ranged from 125,192 bp to 126,105 bp. The average GC content of these chloroplast genomes was 33.78%, with a maximum of 33.9% and a minimum of 33.7% (Table S1). All of these chloroplast genomes are a typical circular structure and contain only one copy of the inverted repeat sequence (IR). Annotation results show that these chloroplast genomes have in common 109 genes, including 75 common protein-coding genes, 4 common ribosomal RNA (rRNA) genes, and 30 common transfer RNA (tRNA) genes (Figure 1). Consistent with the structure of angiosperms, these coding genes are divided into three categories. One is associated with photosynthesis, with 43 genes, of which ndhA, ndhB, petB, petD, and atpF contained an intron. Another group of 57 genes was related to self-replication, including 4 rRNA genes and 30 tRNA genes. The nine genes *rpl2*, *rps12*, *rpoC1*, trnA-UGC, trnG-GCC, trnI-GAU, trnK-UUU, trnL-UAA, and trnV-UAC all have an intron, where *rps12* is a trans-spliced gene. Finally, there four genes for conserved hypothetical chloroplast ORF of unknown function and five genes for other encoded proteins, and we found that clpP contained one intron, *ycf3* contained two introns, and accD had three introns. Among alfalfa chloroplast genomes, *rpl22*, *rpsl6,* and infA genes were absent (Table 1). The chloroplast genome contains a large amount of genetic information, and conservative gene composition is suitable for phylogenetic research in alfalfa. Photosynthesis-related genes and biosynthesis genes are of great significance for the plant resistance to environmental changes.

### 2.2. Comparative Analysis of Genome Structure

**Figure 2:**
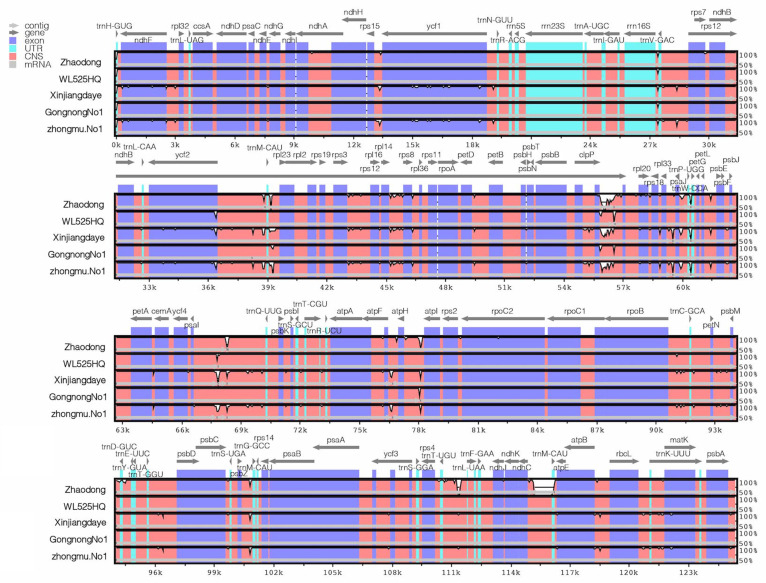
Comparison of chloroplast genome sequences of six varieties of Alfalfa. The ordinate indicates the proportion of agreement (50–100%), the horizontal indicates the chloroplast genome position, and annotated genes are listed at the top. Genomic regions in pink indicate Conservative Non-Coding Sequences (CNSs); exons are in dark blue; rRNAs and tRNAs are in light blue; grey arrows indicate gene orientation.

To better compare chloroplast genomic differences between alfalfa germplasms, mVISTA was used for sequence alignment and assessed sequence identity (Figure S2). *Medicago sativa* Deqin was used as the reference sequence, and the whole sequence consisting of five genomes, including ZhaoDong, WL525HQ, ZhongmuNo1, GongnongNo1, and Xinjiangdaye, was compared (Figure 2). The analyses revealed small differences in these chloroplast genomes, which existed in both coding and non-coding regions, with significant sequence differences visible in non-coding regions in the 36–40 kb, 55–72 kb, and 76–78 kb ranges, and relatively high levels of variation in the *ndhF*, *clpP*, *ycf1*, and ycf2 genes of the coding regions. The results showed that the level of variation in both coding and non-coding regions is higher in cold-tolerant varieties compared to the sensitive variety WL525HQ. The *ycf1*, *matk,* and *ndhF* regions in Xinjiangdaye and ZhongmuNo1 have particularly high levels of variation. Similarly, it was found that *rps12*, *trnW-CCA*, *clpP*, *rps8,* and *psaJ* regions show higher levels of variation in the cold-tolerant varieties. From the perspective of sequence differences, the sequence differences are lowest in the exonic regions, while the conserved non-coding sequences show the highest level of sequence differences. The highly similar sequences in the functional gene regions indicate a high conservation of chloroplast functional genes in alfalfa.

**Figure 3:**
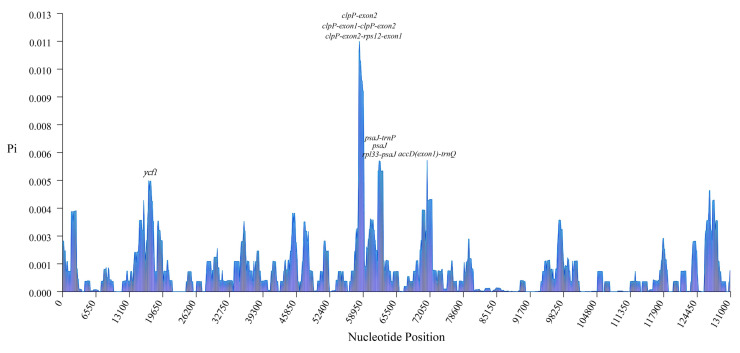
Statistics of nucleotide diversity (Pi) in 231 alfalfa chloroplast genomes. The *X*-axis represents the position of nucleotides; the *Y*-axis represents the nucleotide diversity in each window. Parameters are set to a window length of 1000 bp and a step size of 100 bp.

We also calculated the nucleotide diversity (Pi) values for the chloroplast genomes of the alfalfa germplasm, and the Pi values for the nucleotide diversity of these 231 cpDNAs ranged from 0 to 0.01126 (Table S2, Figure 3). There is high nucleotide diversity in the *clpP*, *psaJ,* and ycf1 genes, and the maximum Pi value is for an exon of the *clpP* gene. We found higher nucleotide diversity in the intergenic region compared to the coding region. We found that the highly variable regions were clustered between 57 kb and 72 kb, which is consistent with the range of regions in our comparative genome analysis, and these spacers were *clpP (exon1)-clpP (exon2)*, *psaJ-trnP*, *clpP (exon2)-rps12 (exon1)*, *rpl33-psaJ*, and *accD (exon1) -trnQ*, with *clpP (exon1)-clpP (exon2)* having the highest nucleotide diversity.

### 2.3. Phylogenetic Tree and Population Genetic Structure Analysis Based on SNPs

**Figure 4:**
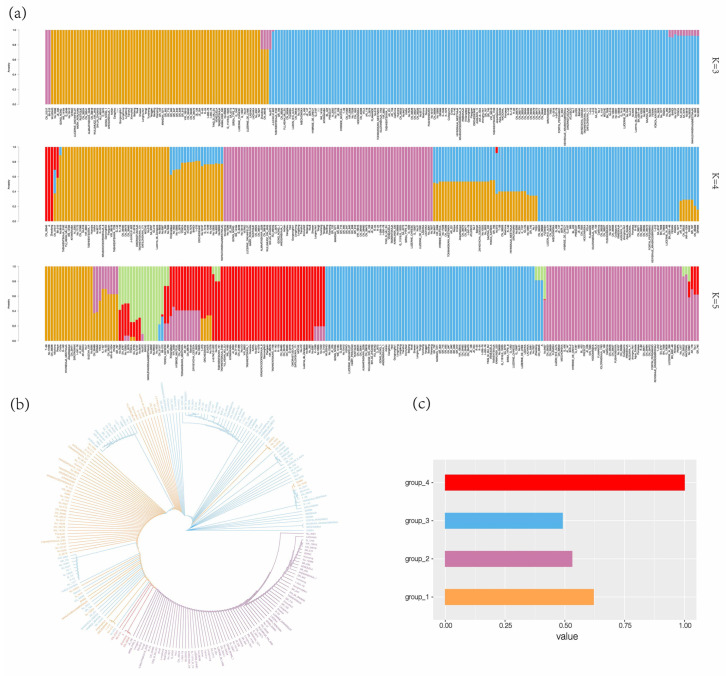
Phylogenetic tree and population genetic structure analysis based on SNP: (**a**) population structure analysis of chloroplast genomes of 231 alfalfa is based on SNP; (**b**) phylogenetic tree of chloroplast genomes of 231 alfalfa are constructed based on SNP; (**c**) the proportion of cold-tolerant varieties in different groups. Each color represents a different group.

To investigate the genetic structure among alfalfa varieties, we extracted SNPs from the alfalfa chloroplast genome. SNP loci were used to construct the ML phylogenetic tree and conduct population structure analyses. Population structure is the existence of varying degrees of genetic relatedness between certain subgroups within species. The cross-validation (CV) error values were calculated for different K values (K = 2 to 9) when alfalfa population structure analysis was performed using admixture. When K = 4, group_1 and group_3 formed group_2 together, and the varieties of group_4 originated from the divergence of group_1. The chloroplast genomes of 231 alfalfa can be divided into four groups: most of the samples are divided into the three largest groups, while the smallest group consisted of just five varieties. We divided the alfalfa population into four major branches by choosing the CV with the lowest value of K = 4 (Figure 4a, Table S4). However, there were differences in the proportion of cold-tolerant varieties among the four groups, and all varieties in group_4 were cold-tolerant. (Figure 4c). Over an extended period of evolution and natural selection, some varieties may have developed stronger cold tolerance genes. The transition from K = 3 to K = 4 suggests that group_2 may have evolved from a common ancestor of group_1 and group_3. The results of constructing a phylogenetic tree based on SNPs indicate that all varieties cluster on one branch (Figure 4b). Group_1 and group_3 have a close genetic relationship. Group_1 has incorporated varieties from group_3, and this result may be attributed to the widespread mutual introduction and cross-fertilization of diverse varieties of alfalfa across different regions. The clustering of group_2 and group_4 is consistent with the results of the population structure analysis. Taken together, the findings suggested that it was better to divide the alfalfa population into four groups.

### 2.4. Haplotype and Phylogenetic Analysis

**Figure 5:**
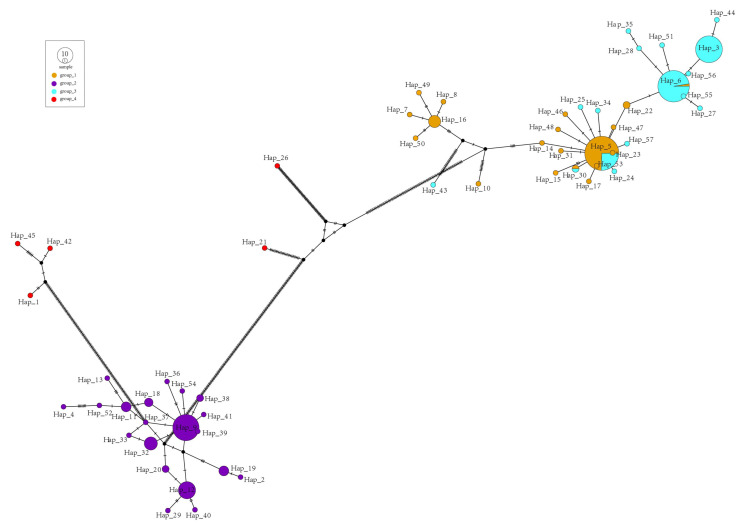
Haplotype network of alfalfa chloroplasts. The size of the circles represents the number of haplotypes. Dots represent putative haplotypes. Group_1 is shown in orange, group_2 in purple, group_3 in blue, and group_4 in red.

We used 231 alfalfa chloroplast genomes for haplotype analysis, which classified 57 haplotypes (Hap_1-Hap_57) (Table S3). Based on the population structure, we divided the alfalfa population into four groups (Figure 5). Group_1 contained 19 haplotypes, and group_2 and group_3 contained 20 and 16 haplotypes. Group_4 had five haplotypes. Haplotype network diagram results show that both group_2 and group_4 form a monophyletic group, while group_1 and group_3 cluster together. Hap_9 is the main haplotype distributed in the group_2 cluster, and it is only distributed in group_2. Most of the haplotypes appear singly. Hap_5, Hap_6, and Hap_30 ae shared haplotypes in group_1 and group_3, and the main distributed haplotypes in group_1 and group_3 are Hap_5 and Hap_6, respectively. This may be caused by the abundant mutual introduction and cross-fertilization of alfalfa between different varieties. Hap_5 has 35 varieties in group_1 and another 12 varieties in group_3. In Hap_6, we find that only one variety is assigned to group_1, while group_3 contains the remaining 39 varieties. Hap_30 contains only one variety that is assigned to both group_1 and group_3. Based on the chloroplast whole genome, we constructed a phylogenetic tree, divided into four groups according to the distribution of haplotypes, and found that the distribution of varieties corresponded to the results of the population structure analysis. 

**Figure 6:**
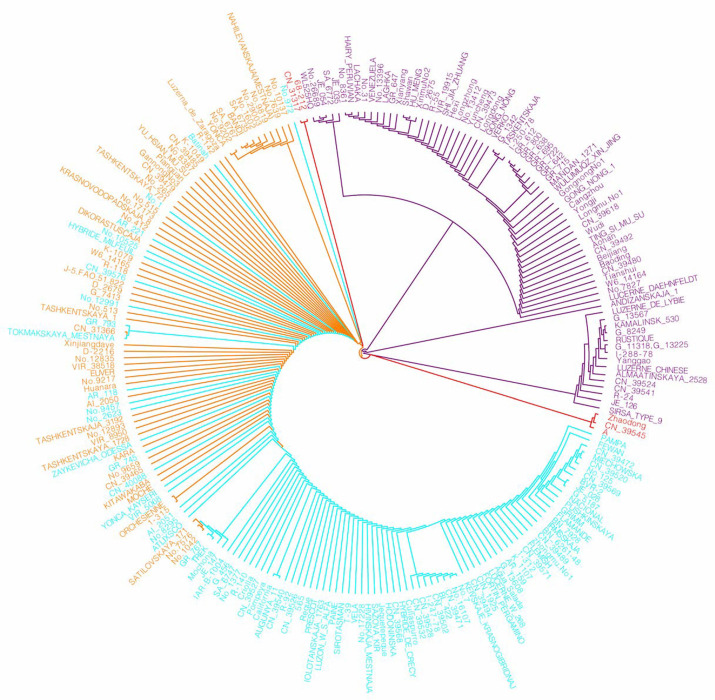
The ML phylogenetic tree based on complete chloroplast genomes. Group_1 is shown in orange, group_2 in purple, group_3 in blue, and group_4 in red.

To explore the phylogenetic relationships among different alfalfa germplasms, we employed the chloroplast genomes of 231 alfalfa to construct an ML phylogenetic tree (Figure 6). The phylogenetic results based on the chloroplast genome sequences differed from the clustering based on the SNP construction mentioned earlier, forming distinct branches for alfalfa. In the comprehensive chloroplast genome phylogenetic tree, one branch included all varieties from group_1 and group_3, while group_2 clustered on another branch. Both the SNP-constructed tree and the chloroplast genome-constructed tree indicated a close affinity between group_1 and group_3, making it challenging to clearly separate them. This could be attributed to genetic infiltration between the varieties of the two groups, suggesting potential genetic admixture or hybridization, leading to individuals with mixed genetic traits. Varieties from group_4 did not cluster together, possibly due to different mutation rates in the coding and non-coding regions of the chloroplast genome. However, the alfalfa population distinctly formed branches, indicating the representativeness of this chloroplast genome in different classification groups of alfalfa.

### 2.5. Response of the Chloroplast Genome of Alfalfa to Cold Stress

**Figure 7:**
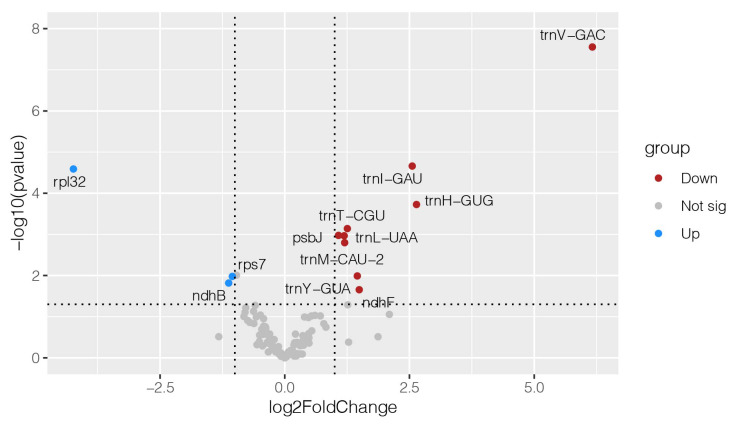
Volcano plot of chloroplast gene expression under cold stress in alfalfa. Two alfalfa varieties, Algonquin and WL525HQ, were studied under cold stress, with three biological replications for each variety. Plot the volcano for the chloroplast genome of alfalfa under cold stress using the average transcriptome data from three biological replicates. The two vertical lines represent genes with a fold change in expression of two or more on the outer side, and the line above indicates a *p*-value less than 0.05.

The chloroplast is one of the most sensitive components to low temperature in plants. Functional genes such as photosynthetic system genes, self-replication genes, and biosynthetic genes encoded by the chloroplast genome play an important role in plant adaptation to temperature changes, and the expression of these genes changes under cold stress. We analyzed six transcriptome data under cold stress using the Algonquin and WL525HQ varieties (Figure 7). Algonquin is cold-tolerant variety, and WL525HQ is a cold-sensitive variety. We found that the expression of genes related to subunits of NADH dehydrogenase in alfalfa was down-regulated in cold-sensitive varieties, e.g., *ndhF*, etc. It is possible that low temperatures affected the normal splicing of the chloroplast gene *ndhB*, which results in abnormal chloroplast development. The expression of genes related to ribosomal subunit proteins was down-regulated in cold-sensitive alfalfa *rpl32* and *rps7*; conversely, these genes were up-regulated in cold-tolerant varieties. This may be a protective mechanism of plant chloroplasts in response to cold stress through ribosome metabolism. We also found that *trnV-GAC*, *trnI-GAU*, and *trnH-GUG* showed mostly significant down-regulation in cold-tolerant varieties. This may be attributed to the adaptive response of the cold-tolerant varieties to cold environments, where the reduction in the expression of trn genes slows down biochemical reactions and protein synthesis, thereby lowering intracellular energy consumption.

**Figure 8:**
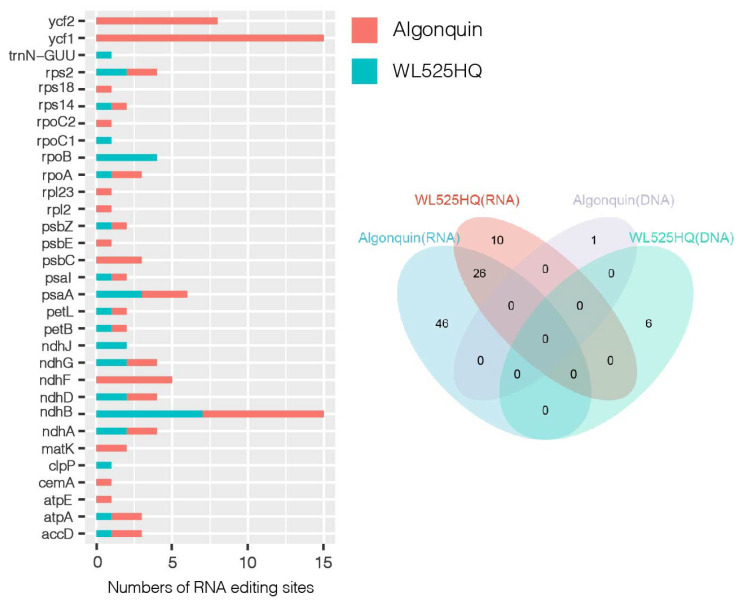
RNA editing events under low-temperature stress in alfalfa varieties: (**a**) analysis of RNA editing efficiency in plastid genes under low-temperature stress in alfalfa; (**b**) number of RNA edits in different cultivars.

We used data from these six transcriptomes to analyze RNA editing events in chloroplasts (Figure 8, Table S5). We identified a total of 31 genes that underwent editing events. We found C to T editing was most common in chloroplast genomes. In the chloroplast genome, we identified a total of eight genes with more than two editing sites. *Ycf1* had the most RNA editing sites, with 15, followed by *ndhB*, *ycf2*, and *ndhF.* Interestingly, we found that the reduced editing efficiency of *ndhF* may promote *ndhF* expression under cold stress. In cold-sensitive varieties, reduced editing sites were observed in *ycf1*, *ycf2,* and *ndhF*. Algonquin and WL525HQ shared 17 editing sites, which were mainly concentrated in *ycf2* and *ndhF* genes. The low temperature led to a decrease in the editing efficiency of these sites, which may slow down the efficiency of electron transfer and thus affect the photosynthesis of chloroplasts. In cold-tolerant varieties, the increased editing efficiency at sites may be a key adaptation to cold environments.

## 3. Discussion

In this study, 231 complete alfalfa chloroplast genomes were assembled and annotated, with an average length of 125,622 bp (Figure 3). These 231 chloroplast genomes have a highly conserved structure, and the GC content of the chloroplast genomes was also high, similar to that previously reported [37,38]. The size of chloroplast genome in higher plants is usually 120 kb to 180 kb. The expansion and contraction of the IR region is one of the important factors affecting the size of the chloroplast genome. The chloroplast genome of alfalfa and most of the legume structures are highly similar to the IR region, and this group is called IRLC [16,39,40]. One example of IR contraction is the IRLC taxon, and it is now believed that large IR contractions may be associated with abnormal genomic rearrangements [41]. It was found that the new IR region is adjacent to *ycf1* and *clpP* in *Medicago minima*, and these two genes may be major regions of genetic recombination due to their high variation [16]. Based on the comparative analysis of chloroplasts and nucleotide diversity, we identified eight highly variable regions, among which *clpP (exon2)* has the highest nucleotide diversity. A large number of studies have used some highly variable regions, such as *clpP (exon1)_psbB*, *rpl33_rps18*, *psaJ_rpl33*, and *ndhF_rpl32,* as DNA barcodes for variety identification [42,43]. In the present study, the intergenic region of *clpP (exon1)-clpP (exon2)*, *psaJ-trnP*, and *clpP (exon2)-rps12 (exon1)* genes is a highly variable fragment, which is expected to be a specific fragment to distinguish alfalfa germplasm resources. In addition to the deletion of the IR region, chloroplast gene structure variation is also affected by the transfer and loss of genes, and it has been found in the study of chloroplast genomes that genes such as *accD*, *rpl23*, *rpl22*, *ycf1*, *ycf2*, *rps16*, *ycf4*, and *infA* have been partially or completely deleted from the chloroplast genomes of leguminous plants, and some genes (e.g., *infA*) have even been lost repeatedly in a variety of plants [44], which was well verified in the chloroplast genome of alfalfa, where deletions of the *rpl22*, *rpsl6*, and *infA* genes were found and could potentially be characteristic of alfalfa varieties.

In higher plants, chloroplasts are organelles with autonomous inheritance and a large amount of genetic information, and chloroplast genome sequences have made significant contributions in exploring the genetic and evolutionary relationships [45,46]. The germplasm resources of alfalfa have similar characteristics, and the evolutionary classification between them is not obvious. Alfalfa is vigorous and can adapt to most geographical environments, which also results in the plasticity of the alfalfa phenotype and genetic diversity, resulting in germplasm diversity. Although a large number of studies have been carried out, it is still difficult to determine the transmission and evolution mechanism of alfalfa [22,23]. We constructed haplotype analyses of 231 alfalfa germplasm resources, and the results were divided into four groups. A total of 88 varieties were closely clustered at the chloroplast genome level. Long et al. divided alfalfa into three clusters based on SNPs in the genome, but these three clusters exhibited that were weakly grouped together [35]. SNPs can be used as molecular markers to study evolutionary relationships; they have high genetic diversity and can be used for variety identification and genetic evolution studies [47,48]. We analyzed the population structure using SNPs from 231 germplasm resources and constructed an exclusive phylogenetic tree of SNPs. The results showed that 231 alfalfa germplasm resources were classified into four groups, and there were still varieties that were closely related. It is presumed that group_1 and group_3 formed group_2 together and continued its performance. Because of the self-incompatibility of alfalfa, genes in different regions are exchanged, forming a highly heterozygous alfalfa. However, the chloroplast genomes of different germplasms are indistinguishable. We found that only the alfalfa varieties in group_4 were cold-tolerant, and other groups were mixed with different phenotypes (Table S4). The population structure clustering information clarified the genetic data divided between different varieties, which was clearer than the results of studies using genomes of alfalfa. Alfalfa involves a large number of genes flowing among species in terms of domestication and adaptation to environmental conditions, resulting in many varieties not able to be classified according to ecotype. It is difficult for the present study to completely solve the problem, but the chloroplast-based classification results provide some useful information for the evolution of alfalfa. 

The chloroplast participates in plant metabolism and some important physiological processes. Cold stress has great effects on chloroplast structure and physiological metabolism [49]. With further research, it was found that cold stress also seriously affects chloroplast protein synthesis and gene expression [50]. The transcriptome data of cold stress can effectively determine how cold temperature affects the expression of plant chloroplast genes, and we found that the expression of *rpl32* and *rps7* is up-regulated in cold-tolerant varieties This suggests that alfalfa may need increased activity of ribosomes to promote protein synthesis and enhance adaptation to cold conditions. Overexpression of ribosomal subunit protein genes in *Arabidopsis thaliana* directly improved its cold tolerance [51]. Photosynthesis is a crucial biological process in plants, providing energy for all life processes. In cold treatment, the expression of *psbJ* is upregulated in sensitive varieties, participating in the capture and conversion of light energy in photosynthesis to promote growth. *PsbS* and *PsbH* have been shown to be upregulated in *Brassica campestris L.* under cold stress, enhancing the role of light-harvesting chlorophyll a/b-binding (*Lhc*) proteins in plant growth and development [52]. Meanwhile, most of the transfer RNA *trnV-GAC*, *trnL-GAU,* and *trnI-GUC* genes were significantly downregulated in cold-tolerant varieties and significantly up-regulated in cold-sensitive varieties. The expression of tRNA genes in cold-tolerant alfalfa was suppressed, which slowed the growth of alfalfa and stored nutrition in winter by reducing the consumption of nutrients in autumn [53,54]. The upregulation of *psbJ* and tRNA genes in sensitive varieties indicates that cold-sensitive alfalfa continues to grow and develop in autumn, and the available sugars for maintaining alfalfa survival decrease in winter, thus reducing the winter survival rate. Compared with cold-sensitive varieties, the *ndhF*, *ycf1,* and *ycf2* genes of cold-tolerant varieties also showed higher RNA editing efficiency under cold stress. There are considerable variations in *ndhF*, *ycf1,* and *ycf2*, and the gene expression of *ndhF* in two varieties is also different. The protein encoded by the unknown function gene ycf1 may involve protein transport as a component of the complex, and the transported protein may be directly related to cold and directly affect the differentiation of varieties. The change in the *ndhF* gene means that it directly affects the adaptability of varieties to cold conditions, which can be used as molecular markers to identify alfalfa varieties. Noncoding regions are also highly variable regions and have higher nucleotide diversity compared to coding regions [55]. Screening of highly variable sequences for the identification of varieties has been successfully used in *Dendrobium* and *Bupleurum* [55,56]. Highly variable regions are suitable for the construction of DNA barcodes *clpP(exon1-clpP(exon2)*, *psaJ-trnP*, *clpP-rps12*, *rpl33-psaJ,* and *ndhB-ndhF* and have great potential in the identification of alfalfa varieties and winter tolerance.

## 4. Materials and Methods

### 4.1. Alfalfa Materials and Plastid Isolation

The 359 alfalfa germplasm resources used in this study were derived from the National Genome Sciences Data Centre (https://ngdc.cncb.ac.cn) (accessed on 15 June 2023). The resequenced data of 137 and 220 core germplasm resources were downloaded from the GSA databases (CRA002917) and (CRA003659) of the NGDC website, respectively. Two alfalfa germplasms, Algonquin and WL525HQ, were grown at Harbin Normal University experiment station. One large seeding was selected from each germplasm, leaves were collected from the selected seedings, and they were rapidly frozen in liquid nitrogen and stored at −80 °C. The samples were sent to BGI Genomics (Shenzhen, China) (http://www.genomics.cn) (accessed on 14 November 2023), where the construction of genomic libraries and sequencing were completed in accordance with the described protocols. The genomic library was constructed with about 420 bp insertion size, and sequencing was performed using the Illumina HiSeq platform with a paired-end sequencing model. All the adapter sequences and low qualitative reads were removed from the raw sequence data; then, the cleaned reads of two alfalfa germplasms were submitted to the GSA database (CRA014561, URL: https://ngdc.cncb.ac.cn/gsa/s/5c2aM2hX) (accessed on 21 January 2024).

### 4.2. Assembly and Annotation of Chloroplast Genome

The chloroplast genome was assembled using GetOrganelle software (version 1.7.5) using *Medicago sativa* Deqin (MN218692) as a reference, and a total of 231 complete chloroplast circular DNAs were obtained after removing redundancy [37]. Collinearity analysis was performed using mummer to verify the accuracy of the assembly [57]. Based on the GenBank format, the file of *Medicago sativa* Deqin was annotated using CPGAVAS2 software (version 2.0) [58], and CPGview-RSG software (http://47.96.249.172:16100/cpgviewer/home) (accessed on 16 November 2023) was used to detect the annotation errors. The sequence length and GC content of the samples were counted by seqkit [58,59]. Finally, the physical mapping of the alfalfa chloroplast genome was performed by the online software CHLOROPLOT (https://irscope.shinyapps.io/Chloroplot/) (accessed on 16 November 2023) [60].

### 4.3. Comparative Analysis of Chloroplast Genome

Sequence differences between the reference chloroplast genome *Medicago sativa Deqin* (MN218692) and the chloroplast genomes of five closely related varieties were compared using the Shuffle-LAGAN model in the online mVISTA tool (https://genome.lbl.gov/vista/index.shtml) (accessed on 18 November 2023) [61]. Nucleic acid diversity was analyzed using DnaSP 6 (DNA Sequence Polymorphism) software (version 6.12.03) with a sliding window length of 1000 bp, a step size of 100 bp, and other parameters set to defaults [62].

### 4.4. Phylogenetic Tree and Haplotype Analysis

The chloroplast genome of 231 alfalfa sequences was aligned using MAFFT software (version 7.487) with default settings [63]. The phylogenetic tree was constructed using IQ-TREE (version 2.0) by selecting the maximum likelihood (ML) technique [64]; the bootstrap value was set as 1000, and the phylogenetic tree was visualized using figtree software (version 1.4.4) [65]. The results of alignment were imported into DnaSP 6 software (version 6.12.03) for the counting of the number of haplotypes (Hap), and POPART software (version 1.7) was used for the construction of the haplotype network diagram [62,66].

### 4.5. Variant Calling

The same reference genome *Medicago sativa* Deqin (MN218692) GenBank format file was selected; the variants of the chloroplast genomes were called using Snippy (https://github.com/tseemann/snippy) (accessed on 21 November 2023), and VCF formats were generated. The SNPs were extracted by VCFtools (version 0.1.17), and Plink (version 1.9) filtered the variant information of SNPs, with the minor allele frequency set higher than 0.05 to output the VCF format file [67,68].

### 4.6. Comparative Analysis Based on SNP

The VCF format file was converted to the phylip format file using Plink [68] and then compared using MAFFT software (version 7.487) [63]. IQ-TREE 2 set the bootstrap value to 1000 to construct the ML phylogenetic tree [64]. The treefile file was imported into figtree (version 1.4.4) to visualize the phylogenetic tree [65]. We then evaluated 231 genetic lineages of alfalfa using ADMIXTURE (version 1.3.0), setting parameters from K = 2 to K = 10, where K = 3, K = 4, and K = 5 used R visualization (version 4.3.0) [69].

### 4.7. Analysis of RNA-Seq and RNA Editing Efficiency

The RNA-seq experiments were performed as described previously [70]. In briefly, seeds of two alfalfa germplasms were selected and germinated on filter paper and then transferred to pots with a mixture of perlite and sand (3:1, *v*/*v*). The seedlings were grown in a chamber and were irrigated with half-strength Hoagland solution every two days. The conditions of the chamber were set as 14 h light conditions at 24 °C (as day) and 10 h dark conditions at 18 °C (as night). Eight weeks later, all alfalfa seedings were transferred to a new chamber at 4 °C. Three hours later, three samples were taken from the cold stress groups for each germplasm; each sample was leaf tissue collected from five individual seedlings. The collected samples were immediately frozen in liquid nitrogen and stored in a −80 °C freezer, and total RNA was extracted from the plants of the two alfalfa germplasms using the RNeasy Plant Mini Kit (Qiagen, Valencia, CA, USA) following the manufacturer’s instructions. The extracted total RNA from six samples was then quantified using the NanoDrop 2000 spectrophotometer (Thermo Fisher, Waltham, MA, USA) and the Agilent 2100 Bioanalyzer (Agilent Technologies, Santa Clara, CA, USA). As previously described, the RNA samples were sent to BGI Genomics (Shenzhen, China) (http://www.genomics.cn) (accessed on 18 June 2023) for RNA-seq, utilizing paired-end sequencing with a read length of 150 bp. All the adapter sequences and low qualitative reads were removed from the raw sequence data; then, the cleaned reads of these samples were submitted to the GSA database (CRA014537, URL: https://ngdc.cncb.ac.cn/gsa/s/G8X9s3Q1) (accessed on 21 January 2024). Clean RNA-seq sequences were mapped to *Medicago sativa* Deqin using Samlon software (version 0.14.2). TPM values were calculated and then visualized using R heatmaps [71]. Quality-controlled reads were aligned to the *Medicago sativa Deqin* chloroplast genome using Burrows-Wheeler Aligner (BWA) software (version 0.7.17) [72] and then spliced with GATK software (version 4.4.0.0) to remove duplicates to mine SNPs for variant information [73]. REDO (version 1.0) detects RNA editing sites in the chloroplast genome based on VCF files from alfalfa [74].

## 5. Conclusions

In this study, we assembled and compared 231 alfalfa chloroplast genomes and identified seven highly variable regions that can be used as molecular markers for alfalfa variety identification. Haplotype, population genetic structure, and phylogenetic tree results revealed that alfalfa can be divided into four groups, but there will still be 88 alfalfa varieties clustered. The results of transcriptome analysis showed that 11 genes were differently expressed in Algonquin and WL525HQ under cold stress, and the results of RNA editing showed that *ndhF*, *ycf1*, and *ycf2* would be edited significantly less efficiently in WL525HQ, which was cold-sensitive; thus, these genes are beneficial for the identification of cold-tolerant varieties. In conclusion, our results provide useful information on the genetic diversity of alfalfa and adaptation to cold stress in the chloroplast genome.

## Figures and Tables

**Table 1:** Chloroplast gene annotation information of alfalfa.

Category	Gene Group	Gene Name
Photosynthesis	Subunits of photosystem I	*psaA*, *psaB*, *psaC*, *psaI*, *psaJ*
	Subunits of photosystem II	*psbA*, *psbB*, *psbC*, *psbD*, *psbE*, *psbF*, *psbH*, *psbI*, *psbJ*, *psbK*, *psbM*, *psbN*, *psbT*, *psbZ*
	Subunits of NADH dehydrogenase	ndhA*, ndhB*, *ndhC*, *ndhD*, *ndhE*, *ndhF*, *ndhG*, *ndhH*, *ndhI*, *ndhJ*, *ndhK*
	Subunits of cytochrome b/f complex	*petA*, *petB*, *petD*, *petG*, *petL*, *petN*
	Subunits of ATP synthase	*atpA*, *atpB*, *atpE*, atpF*, *atpH*, *atpI*
	Large subunit of rubisco	*rbcL*
	Subunits of photochlorophyllide reductase	*-*
Self-replication	Proteins of large ribosomal subunit	*rpl14*, *rpl16*, rpl2*, *rpl20*, *rpl23*, *rpl32*, *rpl33*, *rpl36*
	Proteins of small ribosomal subunit	*rps11*, rps12*, *rps14*, *rps15*, *rps18*, *rps19*, *rps2*, *rps3*, *rps4*, *rps7*, *rps8*
	Subunits of RNA polymerase	*rpoA*, *rpoB*, rpoC1*, *rpoC2*
	Ribosomal RNAs	*rrn16S*, *rrn23S*, *rrn5S*
	Transfer RNAs	trnA-UGC*, *trnC-GCA*, *trnD-GUC*, *trnE-UUC*, *trnF-GAA*, *trnG-GCC*, *trnH-GUG*, trnI-GAU*, trnK-UUU*, *trnL-CAA*, trnL-UAA*, *trnL-UAG*, *trnM-CAU(3)*, *trnN-GUU*, *trnP-UGG*, *trnQ-UUG*, *trnR-ACG*, *trnR-UCU*, *trnS-GCU*, *trnS-GGA*, *trnS-UGA*, trnT-CGU*, *trnT-GGU*, *trnT-UGU*, *trnV-GAC*, *trnW-CCA*, *trnY-GUA*
Other genes	Maturase	*matK*
	Protease	clpP*
	Envelope membrane protein	*cemA*
	Acetyl-CoA carboxylase	accD***
	c-type cytochrome synthesis gene	*ccsA*
	Translation initiation factor	*-*
	other	*-*
Genes of unknown function	Conserved hypothetical chloroplast ORF	*ycf1*, *ycf2*, ycf3**, *ycf4*

Notes: Gene*: gene with one intron; Gene**: gene with two introns; Gene***: gene with three introns; *rps12* indicates a trans-spliced gene.

## Data Availability

The datasets presented in this study can be found in online repositories. The names of the repository/repositories and accession number(s) can be found in the article/Supplementary Material.

## Supplementary Materials

The following supporting information can be downloaded at: https://www.mdpi.com/article/10.3390/ijms25031776/s1.
