# Supplementary figures and images for: Comparative Analysis of Chloroplast Pan-Genomes and Transcriptomics Reveals Cold Adaptation in Medicago sativa
Source: Int J Mol Sci. 2024 Feb 1;25(3):1776. doi: 10.3390/ijms25031776 (PMC10855486; doi:10.3390/ijms25031776)

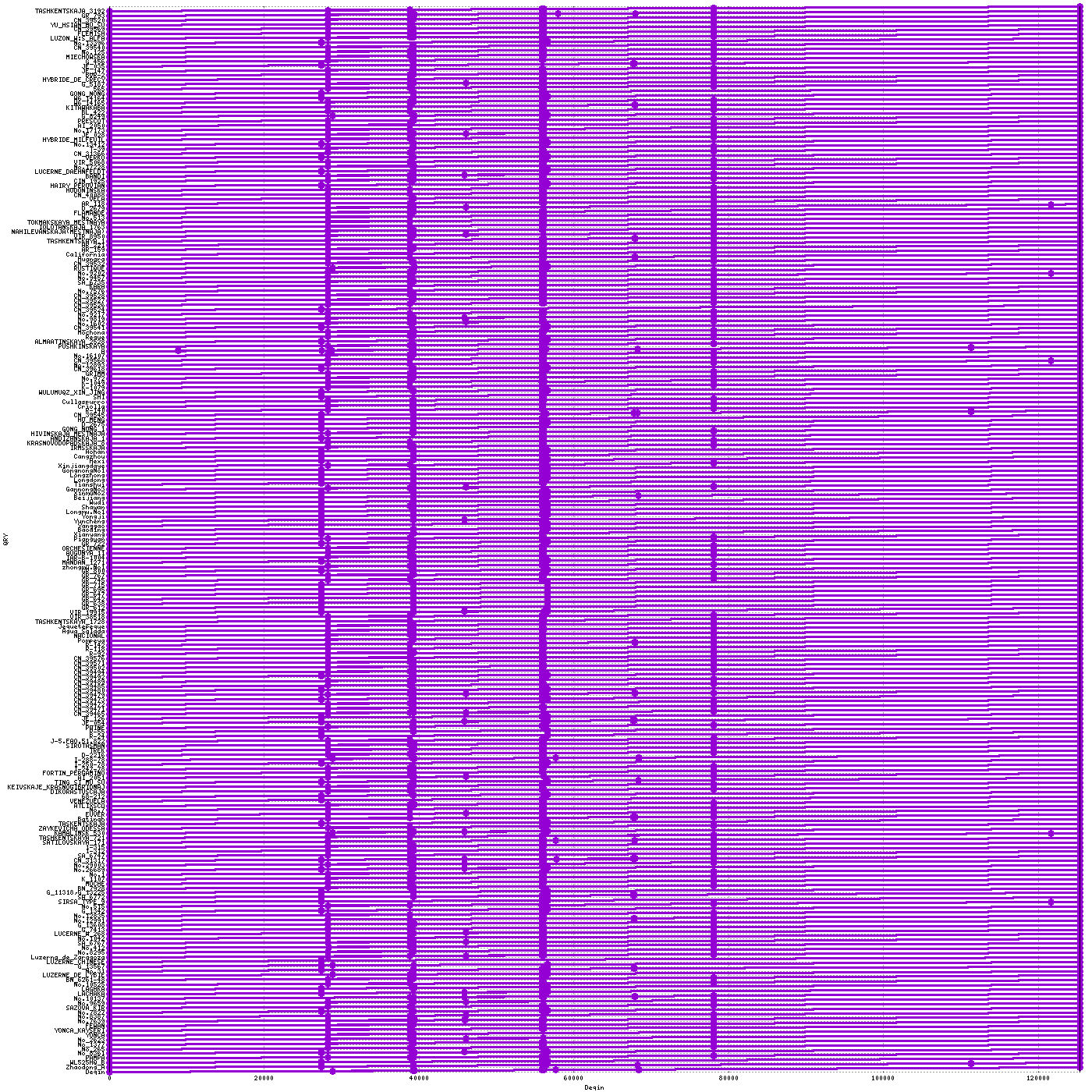

Supplement: Supplementary file 1 [file ijms-25-01776-s001.zip › Figure S1.png]

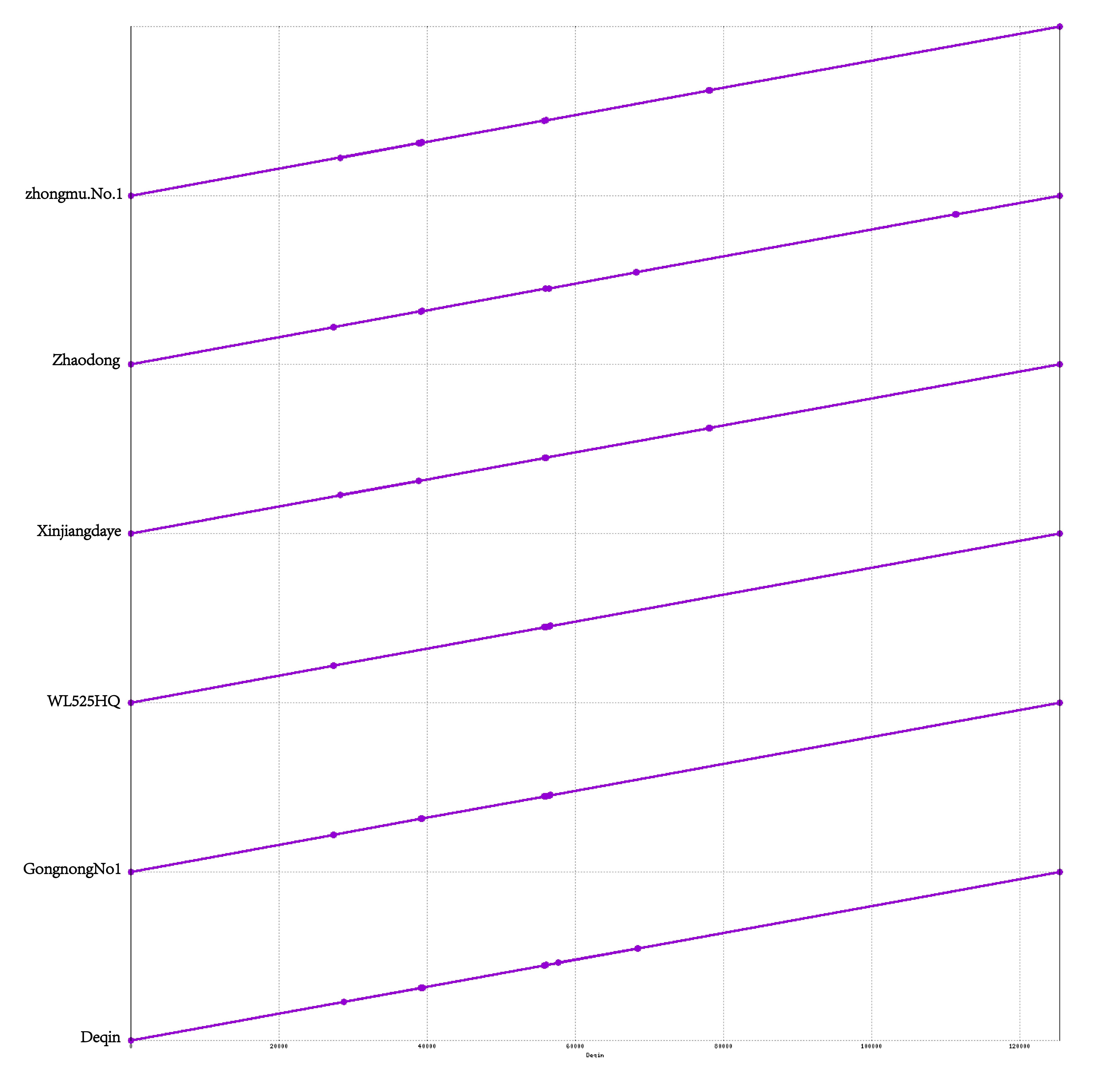

Supplement: Supplementary file 1 [file ijms-25-01776-s001.zip › Figure S2.jpg]
